# Furin-dependent CCL17-fused recombinant toxin controls HTLV-1 infection by targeting and eliminating infected CCR4-expressing cells in vitro and in vivo

**DOI:** 10.1186/s12977-015-0199-8

**Published:** 2015-08-20

**Authors:** Masateru Hiyoshi, Kazu Okuma, Seiji Tateyama, Kazuya Takizawa, Masumichi Saito, Madoka Kuramitsu, Kumiko Araki, Kazuhiro Morishita, Seiji Okada, Naoki Yamamoto, Arya Biragyn, Kazunari Yamaguchi, Isao Hamaguchi

**Affiliations:** Department of Safety Research on Blood and Biological Products, National Institute of Infectious Diseases, Musashimurayama, Tokyo 208-0011 Japan; Medical Facilities Support Department, Micron Inc., Chiyoda-ku, Tokyo, 100-0005 Japan; Division of Tumor and Cellular Biochemistry, Department of Medical Sciences, Faculty of Medicine, University of Miyazaki, Kiyotake, Miyazaki 889-1692 Japan; Division of Hematopoiesis, Center for AIDS Research, Kumamoto University, Kumamoto, 860-0811 Japan; Department of Microbiology, Yong Loo Lin School of Medicine, National University of Singapore, Singapore, 117599 Singapore; Immunoregulation Section, Laboratory of Molecular Biology and Immunology, National Institute on Aging, Baltimore, MD 21224 USA

**Keywords:** HTLV-1, CCR4, CCL17/TARC, *Pseudomonas* exotoxin, Furin

## Abstract

**Background:**

Adult T-cell leukemia (ATL) is caused by human T-cell leukemia virus type 1 (HTLV-1) infection. However, there are no therapies to prevent ATL development in high-risk asymptomatic carriers. To develop a therapy targeting HTLV-1-infected cells that are known to express CCR4 frequently, we tested whether truncated *Pseudomonas* exotoxin (PE38) fused to a CCR4 ligand, CCL17/thymus and activation-regulated chemokine (TARC), selectively eliminates such cells.

**Results:**

Our data show that TARC–PE38 efficiently killed HTLV-1-infected cell lines. It also shrank HTLV-1-associated solid tumors in an infected-cell-engrafted mouse model. In HTLV-1-positive humanized mice, TARC–PE38 markedly inhibited the proliferation of HTLV-1-infected human CD4^+^CD25^+^ or CD4^+^CD25^+^CCR4^+^ cells and reduced the proviral loads (PVLs) in peripheral blood mononuclear cells (PBMCs). Importantly, TARC–PE38 significantly reduced the PVLs in PBMCs obtained from asymptomatic carriers. We show that the cytotoxicity of TARC–PE38 is mediated by the expression of the proprotein convertase, furin. The expression of furin was enhanced in HTLV-1-infected cells and correlated positively with PVLs in HTLV-1-infected individuals, suggesting that infected cells are more susceptible to TARC–PE38 than normal cells.

**Conclusions:**

TARC–PE38 robustly controls HTLV-1 infection by eliminating infected cells in both a CCR4- and furin-dependent manner, indicating the excellent therapeutic potential of TARC–PE38.

**Electronic supplementary material:**

The online version of this article (doi:10.1186/s12977-015-0199-8) contains supplementary material, which is available to authorized users.

## Background

Human T-cell leukemia virus type 1 (HTLV-1) is a human retrovirus that causes HTLV-1-related diseases, including adult T-cell leukemia (ATL) [1–5]. ATL develops in asymptomatic carriers of HTLV-1 after latent infection for at least 20–30 years, with an estimated lifetime risk of 6–7 % for males and 2–3 % for females [6]. A recent national survey in Japan reported at least one million eighty thousand asymptomatic carriers [7]. Although several anti-ATL therapies are available, including chemotherapy [8, 9], ATL is often resistant to these and its prognosis remains poor [10, 11]. The Joint Study on Predisposing Factors of ATL Development (JSPFAD) [12] showed that asymptomatic carriers with a proviral load (PVL) exceeding 4 % (copies/100 cells) constitute a high-risk group in whom ATL selectively develops [13]. Therefore, active interventions, including a prophylactic therapy, are urgently required to prevent the progression to ATL in this high-risk group.

Thymus and activation-regulated chemokine (TARC)/CCL17 and macrophage-derived chemokine (MDC)/CCL22 share CC chemokine receptor 4 (CCR4), which is predominantly expressed on type 2 helper T cells and regulatory T cells (Tregs) [14–17]. Recent studies have demonstrated that CCR4 is frequently expressed in HTLV-1-infected cells, which are predominantly CD4^+^CD25^+^ [18–21]. The expression of MDC is also induced by the HTLV-1 transactivator protein, Tax, in infected cells [22]. The induced MDC seems to attract normal CCR4^+^ T-cells, resulting in the preferential infection of these cells [22] via cell-to-cell contact [23], and maintain a high frequency of functional CCR4^+^FoxP3^+^ Tregs [24].

Recently, a defucosylated humanized anti-CCR4 monoclonal antibody (mAb), mogamulizumab, was developed as an anti-ATL drug [25–30]. This drug specifically binds to CCR4 on ATL cells and exerts antibody-dependent cellular cytotoxicity (ADCC) against the cells by binding to the Fc receptor (FcR) on NK cells. However, although this therapy is effective [30], severe adverse events have been reported [31–33].

In this study, we developed a candidate anti-HTLV-1 therapeutic agent targeting CCR4, with a mode of action that differs from that of mogamulizumab. Our novel agent is TARC-fused truncated 38-kDa *Pseudomonas aeruginosa* exotoxin A (PE38) in which the CD91-binding domain is deleted, designated TARC–PE38 [34]. Full-length PE is known to elicit a cytotoxic effect that is dependent on the intracellular expression of the proprotein convertase, furin [35, 36], when it moves into cells by endocytosis after specifically binding to CD91 on the cell surface via its CD91-binding domain [37, 38]. However, when the CD91-binding domain of PE is switched for another protein-binding domain, the chimeric protein shows a new targeting specificity, dependent on the properties of the switched protein. Thus, TARC–PE38 is expected to specifically bind to CCR4 on target cells via its TARC domain and to exert similar cytotoxic effects on CCR4^+^ cells alone, in a furin-dependent manner. This therapeutic effect on HTLV-1-infected CCR4^+^ cells was tested in vitro and in vivo to determine the drug potential of TARC–PE38 in reducing the risk of ATL development in high-risk asymptomatic carriers.

## Results

### Elimination of HTLV-1-infected CCR4^+^ cells by TARC–PE38

Our previous study showed that TARC-PE38 effectively killed HTLV-1-negative CCR4-expressing T cells, CCRF-CEM, through the apoptosis in vitro and eliminated tumors using this cell line in model mice (in vivo) by the injections subcutaneously and intravenously [34].

**Fig. 1:**
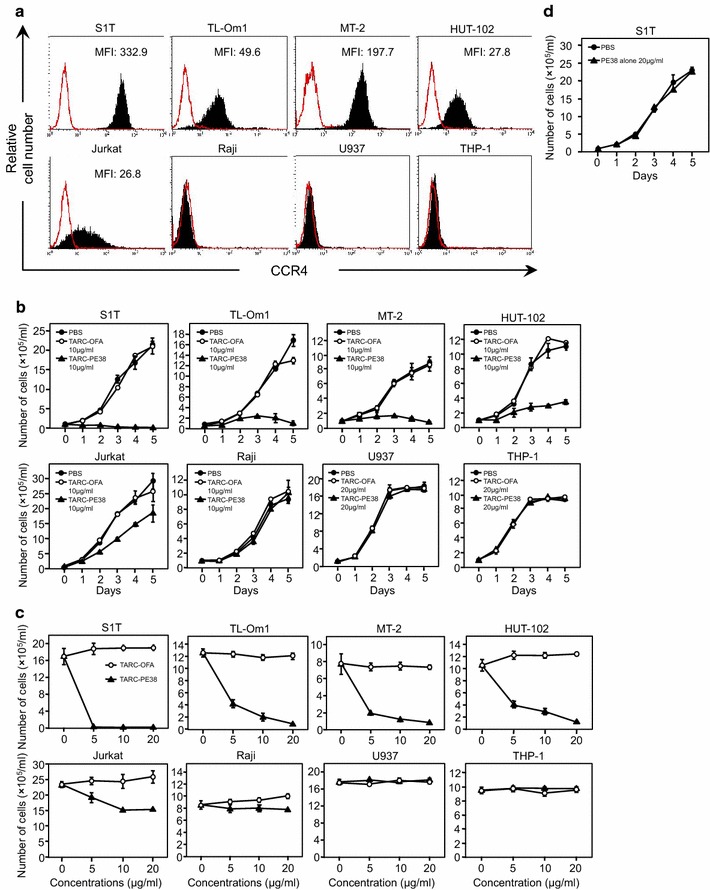
Elimination of HTLV-1-infected cell lines by TARC–PE38. **a** HTLV-1-infected S1T, TL-Om1, MT-2, and HUT-102 cells and uninfected Jurkat, Raji, U937, and THP-1 cells were tested for their expression of CCR4 with flow cytometry without (*solid red line*) or with an anti-CCR4 antibody (*solid line* *filled with black*). MFI, mean fluorescence intensity. **b** To examine the killing effects of TARC–PE38 on the cells described above, the numbers of live cells were counted daily for 5 days after either 10 or 20 μg/ml TARC–PE38 (*closed triangles*), 10 or 20 μg/ml TARC–OFA (*open circles*), or PBS (*closed circles*) was added to each cell line. The data are expressed as means ± SD (×10^5^/ml). **c** To further determine the cytotoxicity of TARC–PE38 in the cells described above, each cell line was incubated with either TARC–PE38 (*closed triangles*) or TARC–OFA (*open circles*) at doses of 0 (PBS only added), 5, 10, or 20 μg/ml for 5 days, and the numbers of live cells were then counted. The data are expressed as means ± SD (×10^5^/ml). **d** To rule out nonspecific effects of PE38, either PBS (*closed circles*) or 20 μg/ml PE38 alone (*closed triangles*) was added to S1T cells. Then the numbers of live cells were counted daily for 5 days, and the data are expressed as means ± SD (×10^5^/ml). These results are representative of four independent experiments

In this study, HTLV-1-infected S1T, TL-Om1, MT-2, and HUT-102 cells were tested to examine the effect of TARC-PE38 on them. These cells were first analyzed with flow cytometry to confirm their cell-surface expression of CCR4 (Fig. 1a). As controls, HTLV-1-uninfected Jurkat, Raji, U937, and THP-1 cells were examined in parallel. As expected, the S1T, TL-Om1, MT-2, and HUT-102 cells expressed CCR4 with the mean fluorescence intensity (MFI) of 332.9, 49.6, 197.7, and 27.8, respectively. The Jurkat cells expressed CCR4 at a lower level (MFI: 26.8) and the Raji, U937, and THP-1 cells expressed no CCR4.

Therefore, we tested the killing effect of TARC–PE38 (10 or 20 μg/ml) on these cells (Fig. 1b). The TARC-fused oncofetal antigen (TARC–OFA; 10 or 20 μg/ml) and phosphate-buffered saline (PBS) were used as controls. Whereas TARC–OFA and PBS did not kill any of the cells tested, TARC–PE38 killed the S1T, TL-Om1, MT-2, and HUT-102 cells efficiently, and the Jurkat cells much less effectively. TARC–PE38 killed no Raji, U937, and THP-1 cells, consistent with their lack of CCR4 expression.

We also examined whether either TARC–PE38 or TARC–OFA (control) killed the same cells in a dose-dependent manner (0–20 μg/ml) (Fig. 1c). Surprisingly, TARC–PE38 killed the S1T cells completely, even at the lowest dose (5 μg/ml), probably because they have the highest cell-surface expression (MFI) of CCR4. TARC–PE38 killed the TL-Om1, MT-2, and HUT-102 cells in a dose-dependent manner. However, even when the dose was increased to 20 μg/ml, the killing effect of TARC–PE38 was only modestly enhanced in the Jurkat cells and not at all in the Raji, U937, and THP-1 cells. TARC–OFA killed none of the cells at any dose tested.

As demonstrated by flow cytometry, the HUT-102 cells expressed CCR4 at a similar MFI to the Jurkat cells (27.8 vs 26.8, respectively), but most whole HUT-102 cells expressed CCR4 like the TL-Om1 cells (almost 100% positive) and unlike the Jurkat cells (lower % positive) (Fig. 1a). Thus, when TARC–PE38 was added to the HUT-102 cells, TARC–PE38 killed the cells more effectively than it killed the Jurkat cells (Fig. 1b, c).

In addition, to rule out nonspecific effects of PE38, we examined whether PE38 alone (no protein fused to PE38) killed CCR4^+^ S1T cells in parallel with PBS. PE38 alone (20 μg/ml) as well as PBS did not kill the S1T cells at all (Fig. 1d). Thus, as neither TARC-OFA, PBS, nor PE-38 alone killed any cells tested, PBS alone was used as a negative control in the following experiments.

Therefore, TARC–PE38 targets CCR4^+^ cells and exerts a cytotoxic effect on HTLV-1-infected cells in vitro, at least in a CCR4-expression-dependent manner.

### Regressive effect of TARC–PE38 on HTLV-1-associated tumor in a mouse model

Next, to examine the in vivo effects of TARC–PE38, we constructed an HTLV-1-associated tumor-bearing mouse model. Because HTLV-1-infected HUT-102 cells are known to form tumors in immunodeficient mice most efficiently among cells tested [39], this cell line was selected to generate this model.

**Fig. 2:**
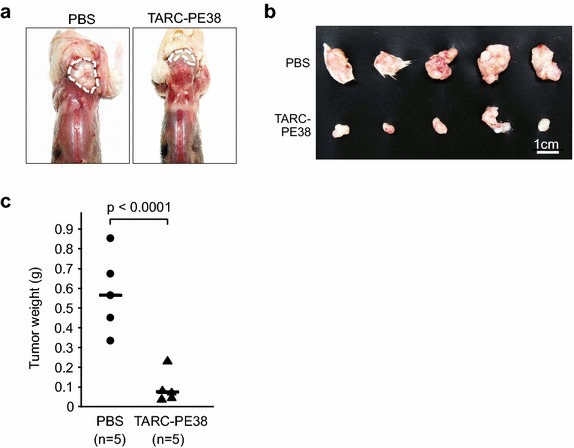
Antitumor effect of TARC–PE38 in a mouse model bearing an HTLV-1-related tumor. **a** HUT-102 cells (2 × 10^7^ cells/mouse) were transplanted into the postauricular regions of 10 NOG mice. Either 25 μg/mouse TARC–PE38 or PBS was then administered to the same region once a day on days 1–5 after transplantation (a total of five times). The mice were observed and sacrificed on day 14 after transplantation to assess the therapeutic effect of TARC–PE38 on the solid tumors grown in the same region. The sizes of the tumors were compared among the mice sacrificed. Representative tumors of mice treated with PBS or TARC–PE38 were photographed with a standard digital camera. The tumors are surrounded by *wavy lines*. **b** All tumors grown in mice treated with PBS (n = 5, *upper line*) or TARC–PE38 (n = 5, *lower line*) were excised from the mice, compared, and photographed in the same way. The *white bar* represents 1 cm. **c** Tumors excised from mice treated with PBS (n = 5, *closed circles*) or TARC–PE38 (n = 5, *closed triangles*) were all weighed (in grams, g), and compared statistically (p < 0.0001). *Bar* represents the mean. These data are representative of more than two independent experiments

In a tumor-bearing mouse model with the HUT-102 cells, TARC–PE38 had a striking regressive effect on the mass of the solid tumors in five mice treated with TARC–PE38 compared with five mice treated with PBS (Fig. 2a, b). The weights of the tumors were more strongly reduced by treatment with TARC–PE38 (mean final weight ~0.1 g) than by treatment with PBS (mean final weight ~0.6 g), and the difference in weight between the groups was statistically significant (p < 0.0001; Fig. 2c).

Therefore, TARC–PE38 efficiently inhibits tumor growth in an HTLV-1-associated tumor-bearing mouse model.

### Therapeutic effect of TARC–PE38 on HTLV-1 infection in humanized mice

**Fig. 3:**
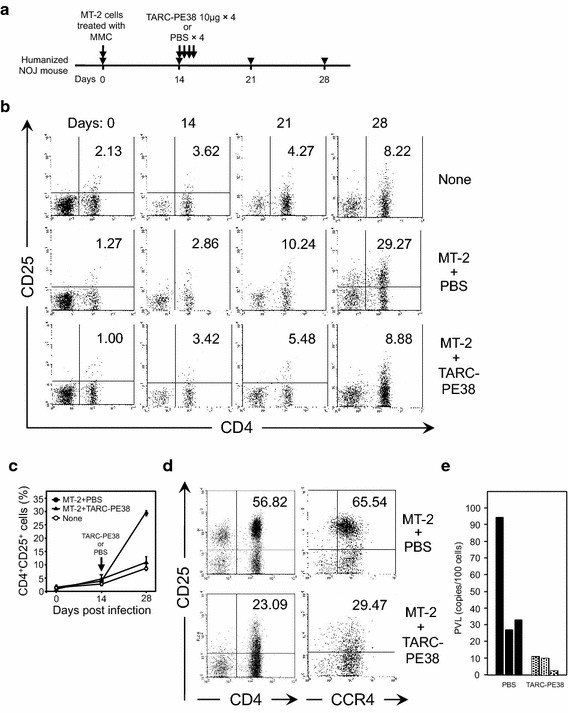
Antiviral effect of TARC–PE38 on HTLV-1 infection in humanized mice. **a** CD34^+^ HSCs were purified from human cord blood and transplanted intrahepatically into newly born NOJ mice. After these mice were humanized, nine mice were inoculated intraperitoneally without (n = 3, PBS only) or with MT-2 cells treated with MMC (n = 6) (day 0). Two weeks later, either 10 μg of TARC–PE38 or PBS was administered to the HTLV-1-infected mice (three mice in each group) intravenously once a day for four successive days (days 14–17). All mice were observed for more than 2 weeks after the administration of TARC–PE38 or PBS, and bled on days 0, 14, 21, 28, and later. **b** To assess the therapeutic effect of TARC–PE38 on HTLV-1-infected cells, peripheral blood was obtained from all the mice described above. The PBMCs were isolated and the frequency of human CD4^+^CD25^+^ cells was determined with flow cytometry. The data are representative of each group as follows: none, uninfected untreated mice; MT-2 + PBS, HTLV-1-infected mice treated with PBS; MT-2 + TARC–PE38, HTLV-1-infected mice treated with TARC–PE38. The *number* indicated in the *upper right* quadrant is the frequency of human CD4^+^CD25^+^ cells (%). **c** The frequencies of human CD4^+^CD25^+^ cells (%) on the indicated days after infection are plotted for each mouse group: none (*open circles*), MT-2 + PBS (*closed circles*), or MT-2 + TARC-PE38 (*closed triangles*). The data are expressed as means ± SD (%). **d** To determine the effect of TARC-PE38 on CD4^+^CD25^+^ or CD4^+^CD25^+^CCR4^+^ cells, PBMCs obtained from each mouse group (MT-2 + PBS or MT-2 + TARC-PE38) after day 28 were checked for the frequency of human CD4^+^CD25^+^ cells (*left panels*) or CD4^+^CD25^+^CCR4^+^ cells (*right panels*) by flow cytometry. The *number* indicated in the *upper right* quadrant is the frequency of human CD4^+^CD25^+^ or CD4^+^CD25^+^CCR4^+^ cells (%). **e** PBMCs obtained on day 28 from MT-2-infected mice treated with PBS (*black bar*) or with TARC–PE38 (*dotted bar*) were also used to measure PVLs with quantitative PCR. The data are presented as copies/100 cells. These results are representative of two independent experiments

To further confirm the in vivo efficacy of TARC-PE38 against HTLV-1, we constructed six HTLV-1-infected and three uninfected, humanized mice. Fourteen days after infection with mitomycin C (MMC)-treated MT-2 cells, we used flow cytometry and quantitative PCR to confirm that no residual MT-2 cells were present in the infected mice (Additional file 1: Figure S1, Additional file 2: Figure S2, Additional file 3: Table S1). As shown in Fig. 3a, either 10 μg of TARC–PE38 per mouse or PBS (control) was administered to three of the six infected mice once a day on days 14–17. All the mice were bled on days 0, 14, 21, 28, and later, and peripheral blood mononuclear cells (PBMCs) were then isolated from the blood.

Using flow cytometry, we tested the frequency of human HTLV-1-infected CD4^+^CD25^+^ cells in human CD45^+^ cells of the PBMCs (Fig. 3b, c). Because binding of TARC to CCR4 on cells induces internalization (down-regulation) of CCR4 via endocytosis of a TARC-CCR4 complex [34, 40], reduction of CCR4^+^ cells by treatment with TARC-PE38 reflects disappearance of CCR4 but may not exactly reflect elimination of CCR4^+^ cells (the killing effect of TARC-PE38 on CCR4^+^ cells). Therefore, to evaluate the killing effect of TARC-PE38 on infected (CD4^+^CD25^+^CCR4^+^) cells accurately, we checked CD4^+^CD25^+^ cells rather than CCR4^+^ cells. In the MT-2-infected mice treated with TARC–PE38, the increase in the frequency of HTLV-1-infected cells was markedly inhibited compared with that in the MT-2-infected mice treated with PBS (mean 5.48 vs 10.24 %, respectively, on day 21, and 8.88 vs 29.27 %, respectively, on day 28) and almost the same as that in the uninfected mice (8.22 % on day 28).

However, because in spite of TARC-induced internalization of CCR4, TARC-PE38 undergoes degradation after administration to mice in the humanized mouse model, the internalization of CCR4 is presumably not persistent during the long observation in vivo. Thus, data for CCR4^+^ cells in the humanized mouse model could be still important and supportive to evaluate the therapeutic effect of TARC-PE38, and indicated significant CCR4^+^ cell reduction by treatment with TARC-PE38 (Additional file 4: Figure S3). It was also demonstrated after day 28 that a population of CD4^+^CD25^+^ cells (Fig. 3d, left panels) or CD4^+^CD25^+^CCR4^+^ cells (Fig. 3d, right panels) completely disappeared by treatment with TARC-PE38, indicating that the treatment selectively targets CD25^+^ (infected) cells, not CD25^−^ (uninfected) cells, especially among CCR4^+^ cells.

We also measured the PVLs with quantitative PCR in PBMCs collected on day 28 from each mouse treated with TARC–PE38 or PBS. Consistent with the above results, the PVLs (<10 copies/100 cells) in the PBMCs from mice treated with TARC–PE38 were significantly lower than those (around 30–90 copies/100 cells) in mice treated with PBS (Fig. 3e).

Thus, TARC–PE38 exerts a potent therapeutic effect on HTLV-1 infection in a humanized mouse model.

### TARC–PE38 reduces PVLs in PBMCs from asymptomatic carriers

We also examined the therapeutic effect of TARC–PE38 on primary HTLV-1-infected cells. Because in this study we mainly focused on the therapeutic potential of TARC-PE38 to prevent the development of ATL for high-risk asymptomatic HTLV-1 carriers, not for ATL patients, we used PBMCs obtained from asymptomatic carriers alone in this experiment.

**Fig. 4:**
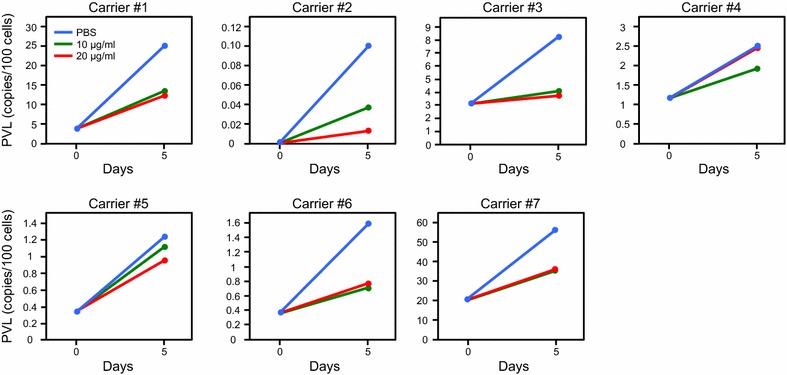
Reduction in PVLs in PBMCs from asymptomatic carriers by TARC–PE38. PBMCs obtained from seven asymptomatic carriers (carriers #1–7) were incubated for 5 days in the absence (0 μg/ml, PBS only:* blue line*) or presence of 10 (*green line*) or 20 μg/ml TARC–PE38 (*red line*). The PVL in each culture was then measured with quantitative PCR. The data are presented as copies/100 cells. These results are representative of more than two independent experiments

PBMCs obtained from seven asymptomatic carriers were incubated with 0 (PBS), 10, or 20 μg/ml TARC–PE38 in the presence of interleukin 2 (IL2) for 5 days. The genomic DNA of the harvested PBMCs was then purified and used to measure the PVLs with quantitative PCR. Because of TARC-induced internalization of CCR4, reduction of CCR4^+^ cells by treatment with TARC-PE38 may not exactly reflect elimination of CCR4^+^ cells (the killing effect of TARC-PE38 on CCR4^+^ cells) again. We therefore checked PVLs rather than CCR4^+^ cells to evaluate the killing effect of TARC-PE38 on infected cells accurately. As shown in Fig. 4, the PVLs all increased after the incubation as compared to those before the incubation. However, the PVLs in five of seven carriers tested definitely decreased after incubation with TARC–PE38 compared with those after incubation with PBS, probably by killing HTLV-1-infected primary cells. This indicated that TARC–PE38 can effectively reduce the PVLs increasing during the active viral production.

### Furin-dependent cytotoxic effect of TARC–PE38 on HTLV-1-infected cells

Because TARC–PE38 targets CCR4, it can attack normal CCR4^+^ cells. Thus, we compared a gene expression involved in the susceptibility to TARC–PE38 between HTLV-1-infected and uninfected CCR4^+^ cells.

Because full-length PE is cleaved within cells by the proprotein convertase, furin, which mediates its cytotoxicity [35, 36], we investigated the expression of furin. If there were a difference in the intracellular expression of furin by HTLV-1-infected and uninfected CCR4^+^ cells, it may affect the killing effect of TARC–PE38 on these cells.

**Fig. 5:**
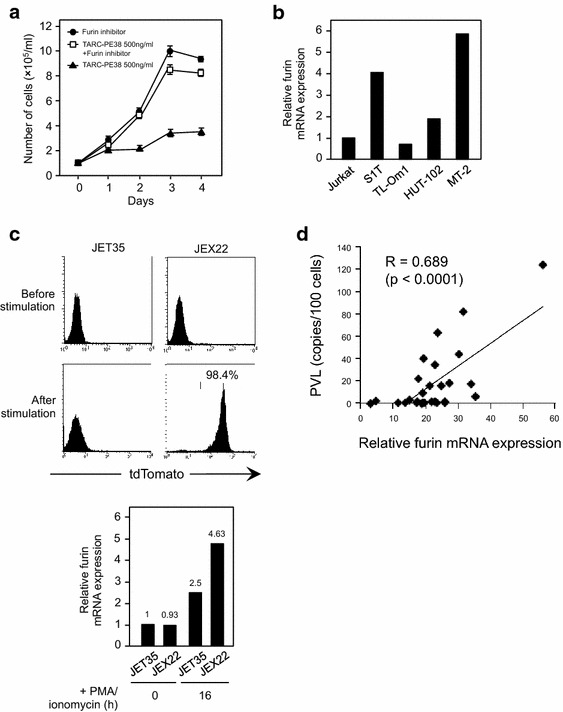
Furin-dependent activity of TARC–PE38 and increase in furin expression by HTLV-1 infection. **a** To test whether the effect of TARC–PE38 is dependent on furin expression, S1T cells were incubated with 500 ng/ml TARC–PE38 only (*closed triangles*), 500 ng/ml TARC–PE38 and a furin inhibitor (*open squares*), or the furin inhibitor only (*closed circles*) for 4 days. The numbers of live cells were counted daily to assess cell viability. The data are presented as means ± SD (×10^5^/ml). **b** Furin mRNA expression levels were measured with quantitative RT–PCR in HTLV-1-infected S1T, TL-Om1, HUT-102, and MT-2 cells and uninfected Jurkat cells. The data are presented as the expression level determined in each cell line relative to that in Jurkat cells. **c** The frequencies of tdTomato-positive cells in parental uninfected JET35 cells and HTLV-1-infected JEX22 cells before and 16 h after stimulation with PMA/ionomycin were analyzed with flow cytometry (*upper panels*) and the expression levels of furin were measured with quantitative RT–PCR (*lower panel*). The frequency in stimulated JEX22 cells is presented as a percentage (%) and the expression level determined in each cell line is relative to that in Jurkat cells. As for the stimulated JEX22 cells, only tdTomato-positive HTLV-1-producing cells were isolated and used for the assays. **d** PBMCs obtained from 29 HTLV-1-infected individuals were used to examine the relationship between furin expression and PVLs. The furin mRNA expression levels and PVLs in these PBMCs were determined with quantitative RT–PCR and quantitative PCR, respectively. Each furin mRNA expression level is presented as the level determined in each sample relative to that in Jurkat cells. PVLs determined in the samples are presented as copies/100 cells. The correlation between the two parameters was then investigated statistically (R = 0.689, p < 0.0001)

To examine whether the cytotoxic activity of TARC–PE38 is mediated by the expression of furin, we tested whether a furin inhibitor blocked the killing effect of TARC–PE38 in the S1T cells. Whereas the cells incubated with TARC–PE38 alone were killed expectedly, the cells incubated with both TARC–PE38 and the furin inhibitor grew well, at a similar rate to cells incubated with the furin inhibitor alone (Fig. 5a). Therefore, the furin inhibitor definitely blocked the effect of TARC–PE38, even in the S1T cells which are most susceptible to TARC–PE38 of all the cells tested (as shown in Fig. 1b, c), confirming that the effect of TARC–PE38 is furin-dependent.

Therefore, we quantified the expression of furin mRNA with quantitative RT–PCR in HTLV-1-infected S1T, TL-Om1, HUT-102, and MT-2 cells and in uninfected Jurkat cells, all CCR4^+^ cells (shown in Fig. 1a). The relative expression levels in the S1T, HUT-102, and MT-2 cells were around four-, two-, and six-fold higher, respectively, than that in the Jurkat cells, whereas that in the TL-Om1 cells was almost the same (Fig. 5b). These data indicated that most HTLV-1-infected cells express higher levels of furin than uninfected cells.

Regardless of the similar expression of the furin in TL-Om1 and Jurkat cells, TL-Om1 cells were clearly more susceptible to TARC-PE38 than Jurkat cells (shown in Fig. 1b, c). This suggests that when the furin is expressed similarly, the susceptibility to TARC-PE38 relies on the expression levels of CCR4 alone, as in fact those of CCR4 (MFI and/or percentage positive cells) were definitely higher in TL-Om1 cells than in Jurkat cells (shown in Fig. 1a).

### Intracellular furin expression is increased by HTLV-1 infection/production

We then sought to clarify whether the intracellular expression of furin is enhanced by HTLV-1 infection. We compared two CCR4^+^ Jurkat-based cell lines, uninfected parental JET35 cells and HTLV-1-infected JEX22 cells which produce HTLV-1 upon stimulation with phorbol 12-myristate 13-acetate (PMA)/ionomycin and express tdTomato (around 27 % positive when analyzed with flow cytometry), dependent on Tax expression. In these cells, we determined the frequencies of tdTomato-positive cells and the expression levels of furin mRNA with flow cytometry and quantitative RT–PCR, respectively, before and after the cells were stimulated. A flow-cytometric analysis showed that the JET35 and JEX22 cells expressed almost no tdTomato before stimulation. The stimulated JET35 cells were tdTomato-negative, whereas 98.4 % of the stimulated JEX22 cells were tdTomato-positive after sorting (Fig. 5c, upper panels). Quantitative RT–PCR showed the relative expression levels in each type of cell compared with that in Jurkat cells (Fig. 5c, lower panel). Interestingly, before stimulation, the expression of furin in HTLV-1-nonproducing JEX22 cells was almost the same as that in the JET35 cells (0.93 vs 1, respectively). However, after stimulation, the expression of furin in the HTLV-1-producing JEX22 cells (sorted above) was around two-fold higher than that in the JET35 cells (4.63 vs 2.5, respectively). Also the expression of furin in the same stimulated JEX22 cells was around five-fold higher than that in the non-stimulated JET35 or JEX22 cells (4.63 vs 1 or 0.93, respectively). These data demonstrated that HTLV-1 infection/production enhances the intracellular expression of furin.

To support this finding, we checked whether there was a positive correlation between PVLs and the expression of furin mRNA in PBMCs collected from 29 HTLV-1-infected individuals (Additional file 5: Table S2). When PVLs and the expression of furin mRNA were measured in PBMCs with quantitative PCR and quantitative RT–PCR, respectively, there was a clear positive correlation between them (R = 0.689, p < 0.0001) (Fig. 5d).

These results strongly suggest that TARC–PE38 eliminates HTLV-1-infected CCR4^+^ cells more effectively than normal CCR4^+^ cells in a furin-expression-dependent manner.

## Discussion

Approximately 20–30 % of asymptomatic carriers are in the high-risk group, with >4 % (copies/100 cells) PVL in Japan [13]. To block the development of ATL in this group, the PVL must be controlled. Therefore, in this study, we focused on HTLV-1-infected CCR4^+^ cells carrying the provirus, to develop a new antiviral agent.

A defucosylated humanized anti-CCR4 mAb, mogamulizumab, was recently developed as a CCR4-targeting immunotherapy for ATL [25–30]. This study confirmed that a CCR4-targeting therapy against ATL cells is convincing and reliable. However, the anti-CCR4 mAb therapy induces several serious adverse effects, including Stevens–Johnson syndrome/toxic epidermal necrolysis, when normal CCR4^+^ Tregs are attacked [31–33].

As an “immunotoxin” (toxin fused to a ligand) [41–43] against ATL cells strongly expressing CD25 (IL2 receptor α chain), anti-Tac (Fv)-PE38/LMB-2, in which the Fv portion of an anti-CD25 antibody is fused to PE38, has been developed by other investigators [44, 45], but its therapeutic effect on ATL was limited [44]. Therefore, we developed TARC–PE38 targeting CCR4 as a novel candidate anti-HTLV-1 drug.

When we examined the therapeutic effect of TARC–PE38 on primary HTLV-1-infected cells, responders (carriers #1, 2, 3, 6, and 7) and non-responders (carriers #4 and 5) to TARCPE38 could be clearly identified among the asymptomatic carriers. This difference in response to TARC-PE38 is probably, at least in part, due to the expression levels of CCR4 at the cell surface of PBMCs obtained.

TARC–PE38 has several major advantages. (1) Although the anti-CCR4 mAb functionally requires the ADCC activity mediated by NK cells, TARC–PE38 exerts a cytotoxic effect by itself, independent of ADCC activity. Therefore, TARC–PE38 can even be used in the absence of functional NK cells in immunocompromised hosts. (2) The anti-CCR4 mAb functions by binding to FcR on NK cells, but does not kill CCR4-expressing ATL cells uniformly in vitro, suggesting that the function of the anti-CCR4 mAb may be affected by polymorphism of FcR [26]. In fact, FcR polymorphisms have been shown to impact the ADCC activity of other antibody-treatments [46, 47]. However, since TARC–PE38 does not need to bind to FcR to function, the function of TARC–PE38 is not affected by FcR polymorphisms. (3) The anti-CCR4 mAb attacks normal CCR4^+^ cells in the same way as it attacks CCR4^+^ ATL cells, whereas TARC–PE38 seems to attack HTLV-1-infected CCR4^+^ cells more effectively than it attacks normal CCR4^+^ cells. This advantage depends on the upregulation of furin expression in the infected cells, and may enhance the therapeutic effect additively/synergistically while reducing the severe adverse effects of the CCR4-targeting therapy.

The PE38 protein is immunogenic in the human body. Because of this immunogenicity there is a limitation in doses given and repeated administrations of PE38-fused proteins to patients with normal immune systems. However, it was recently reported that silencing of T-cell epitopes in PE38 could diminish its immunogenicity [48]. Such a modification of PE38 would be very helpful in clinical applications of TARC-PE38.

The expression of the proprotein convertase, furin, is essential for the maintenance of the peripheral immune tolerance mediated by Tregs [49]. It has recently been reported that HTLV-1 primarily infects Tregs in patients with HTLV-1-associated myelopathy/tropical spastic paraparesis (HAM/TSP) [21]. Therefore, HTLV-1 may take advantage of furin to enhance Treg functions by the preferential infection and expansion of Tregs. This idea is supported by the results of this study, which demonstrate both enhanced furin expression in HTLV-1-infected cells and a positive correlation between furin mRNA expression and PVLs in HTLV-1-infected individuals. However, the molecular mechanism underlying the increase in furin expression during HTLV-1 infection remains to be clarified. The expression of furin is not elevated by the induced expression of HTLV-1 Tax in JPX-9 cells [50] (Additional file 6: Figure S4).

A recent study has also shown that furin mRNA expression in the peripheral blood correlates with chronic hepatitis B virus (HBV) infection, suggesting the participation of furin in Treg-mediated immune suppressive mechanisms during HBV infection [51]. This supports our finding that furin mRNA expression correlates with HTLV-1 infection. These observations during HTLV-1 and HBV infections may reflect a common survival strategy of viruses, through furin-mediated Treg function, to avoid the human immune response to viral infection. Because furin is probably involved in processing the HTLV-1 envelope glycoproteins [52], which play an important role in viral entry into host cells [53, 54], the enhanced expression of furin may also facilitate virus production.

## Conclusions

In this study, we demonstrated the robust therapeutic effects of TARC–PE38 on HTLV-1 infection in vitro and in vivo. We also report that TARC–PE38 may function more efficiently against HTLV-1-infected cells than against normal cells through the proprotein-converting (toxin-cleavage) activity provided by the HTLV-1-induced increase in furin expression. Therefore, because the therapeutic effects of TARC–PE38 depend on the expression of both CCR4 and furin, the effects mediated by the expression of each of these proteins may be additive, synergistic, and/or compensative. Undoubtedly, the strong expression of these proteins in HTLV-1-infected cells should greatly enhance the efficacy of TARC–PE38. Collectively, our results indicate that TARC–PE38 is a useful and promising candidate therapeutic agent for controlling HTLV-1 infection, and therefore has significant prophylactic potential in preventing the development of ATL in the high-risk asymptomatic carriers of HTLV-1.

## Methods

### Cells

The Jurkat and Raji cells are HTLV-1-negative human T-cell and B-cell lines, respectively. The U937 and THP-1 cells are HTLV-1-negative human monocytic cell lines. The S1T, TL-Om1, MT-2, and HUT-102 cells are HTLV-1-infected human T-cell lines [55–58]. The JET35 cells are indicator Jurkat cells stably transfected with a plasmid encoding the tdTomato under the control of the Tax response element. The JEX22 cells are the JET35 cells infected with the HTLV-1 molecular clone pX1 MT-M [59]. When stimulated with 50 ng/ml PMA and 1 μM ionomycin for 16–24 h, The JEX22 cells produce HTLV-1 and simultaneously express tdTomato in response to the Tax expression.

Human peripheral blood obtained from HTLV-1-infected individuals including asymptomatic carriers was provided by JSPFAD. PBMCs were isolated from the blood with Ficoll–Histopaque (Sigma-Aldrich, St. Louis, MO, USA) density gradient centrifugation. To test the effect of TARC-PE38, these primary cells were incubated in the presence or absence of TARC-PE38, supplemented with 20 U/ml recombinant human IL2 (Shionogi Pharmaceutical, Osaka, Japan) for 5 days.

All research protocols were approved by the Institutional Review Boards of the National Institute of Infectious Diseases (Tokyo, Japan) before the start of the study.

### Mice

NOD/SCID IL2-receptor-γ-chain-knockout (NOG) and NOD/SCID Jak3-knockout (NOJ) mice were obtained from the Central Institute for Experimental Animals (Kawasaki, Japan) and Kumamoto University (Kumamoto, Japan), respectively [60, 61]. These mice were maintained under specific-pathogen-free conditions at the Animal Center of the National Institute of Infectious Diseases. Research protocols for the animal experiments were approved by the Animal Center and the ethics committee at the National Institute of Infectious Diseases, and they were performed according to institutional guidelines for the experimental use of animals.

### Production of TARC-fused recombinant proteins

The generation of TARC–PE38 and TARC-OFA, used as a control, has been described previously [34]. PE38 alone (without TARC) was produced as well.

Because TARC-PE38 has a tag at the C terminus to purify this protein, we also generated a different form of TARC-PE38, which the same tag is conjugated with at the N terminus to make the C terminus ending in REDL(K) or KDEL that is needed for the toxin activity of PE [62, 63]. However, this form was not used in this study, due to its low activity relative to that of TARC-PE38 (data not shown).

### Cell viability assay

Approximately 1 × 10^5^ cells were seeded in each well of a 12-well plate. Different doses (0–20 μg/ml) of TARC–PE38 were then added to the wells containing the target cells, in triplicate. As controls, other cells were treated in parallel with the same volume of PBS, the same doses of TARC–OFA, and/or the same doses of PE38 alone.

To determine whether the effect of TARC–PE38 is dependent on furin, a furin inhibitor (Furin Inhibitor I, Calbiochem, Billerica, MA, USA) (75 μM) was added to S1T cells seeded as described above in the presence or absence of TARC–PE38 (500 ng/ml). As an additional control, TARC–PE38 alone was added to the cells in parallel.

These mixtures were incubated for 4–5 days, and cell viability was quantified daily with the standard Trypan-blue exclusion method by counting the number of live cells (×10^5^/ml) in each well under a CK40 microscope (Olympus, Tokyo, Japan).

### Flow-cytometric analysis

In preparation for cell-surface staining, aliquots (~5 × 10^5^) of cells were initially incubated with an FcR-blocking reagent (Miltenyi Biotec, Gladbach, Germany) at 4 °C for 15 min. Cell lines were then stained with or without fluorescein isothiocyanate-labeled anti-CCR4 antibody (R&D Systems, Minneapolis, MN, USA) at 4 °C for 30 min. PBMCs were incubated with or without fluorescein isothiocyanate-labeled anti-human CD4 antibody, phycoerythrin-labeled anti-human CD25 antibody, and allophycocyanin-labeled anti-human CCR4 or CD45 antibody (eBioscience, San Diego, CA, USA) at 4 °C for 30 min.

The stained samples were fixed in 4 % paraformaldehyde (Wako, Osaka, Japan) and analyzed with a FACSCalibur flow cytometer using the CellQuest software (BD Pharmingen, Franklin Lakes, NJ, USA).

### Quantitation of HTLV-1 proviral load

To determine the PVL, the copy numbers of proviral DNA were measured with real-time PCR using the 7500 Fast Real-Time PCR System (Applied Biosystems, Foster City, CA, USA). Genomic DNA (~200 ng) isolated from PBMCs with a QIAamp DNA Blood Mini Kit (Qiagen, Hilden, Germany) was used as the PCR template. Quantitative real-time PCR was performed in duplicate to amplify the *pX* region of the HTLV-1 provirus. The copy numbers of the human *RNase P* gene were measured in parallel as an internal control. The primers and probe for *RNase P* were purchased from Applied Biosystems. Those for HTLV-1 *pX* were: forward primer pX2-S (5′-CGGATACCCAGTCTACGTGTT-3′), reverse primer pX2-AS (5′-CAGTAGGGCGTGACGATGTA-3′), and FAM-labeled pX2 probe (5′-CTGTGTACAAGGCGACTGGTGCC-3′) [64]. The copy number of *pX* in each sample was determined relative to that in TL-Om1 cells. Finally, the PVLs of the tested cells were calculated as ([2 × copies of *pX*]/[copies of *RNase P*]) × 100 and expressed as copies/100 cells.

### Quantitation of furin expression

Quantitative real-time reverse transcription (RT)–PCR with the same system as described above was used to determine the mRNA levels of furin, in various cells. After JEX22 cells were stimulated for 16 h, tdTomato-positive cells were sorted with JSAN (Bay Bioscience, Kobe, Japan) and used. After total RNA was isolated from the cells and purified with Isogen (Wako, Osaka, Japan), quantitative real-time RT–PCR was performed, in duplicate, to quantify both *FURIN* and *GAPDH* (internal control). The primer set for the *FURIN* was: forward primer 5′-TTCACCAACACGTGGGCTG-3′ and reverse primer 5′-CGATGCCAGAAGTGGTAATAGTCC-3′. The primer set for *GAPDH* was: forward primer 5′-ACCCACTCCTCCACCTTTGA-3′ and reverse primer 5′-CTGTTGCTGTAGCCAAATTCGTT-3′. The furin mRNA level in each sample was calculated as 2^−(Ct[furin] − Ct[GAPDH])^ and expressed as the fold difference relative to that in Jurkat cells.

### Administration of TARC–PE38 to a tumor-bearing mouse model

Ten NOG mice were anesthetized with pentobarbital sodium (Kyoritsuseiyaku, Tokyo, Japan) and the postauricular region of each mouse was inoculated subcutaneously with 2 × 10^7^ HUT-102 cells [39]. After the cells were grown for ~24 h, either 25 μg of TARC–PE38 per mouse or the same volume of PBS (control) was administered to the same region in five mice per group daily for 5 days. All the mice were sacrificed 2 weeks after inoculation with the cells and the subcutaneous tumors were excised, weighed, and photographed with a standard digital camera (Fujifilm, Tokyo, Japan).

### Establishment of humanized mice

Mononuclear cells were isolated from human cord blood (provided by Tokyo Cord Blood Bank, Tokyo, Japan) with Ficoll–Histopaque density gradient centrifugation. Human CD34^+^ hematopoietic stem cells (HSCs) were then purified with a Magnetic-Activated Cell Sorting Direct CD34 Progenitor Cell Isolation Kit (Miltenyi Biotec), according to the manufacturer’s instructions. After the cells were enriched twice, more than 85 % of the HSCs obtained were CD34^+^. These cells (1–2 × 10^5^ cells per mouse) were immediately transplanted intrahepatically into newborn NOJ mice [61]. At about 3 months after transplantation, the frequency of human T cells in PBMCs was checked with flow cytometry to determine whether the mice were humanized.

### Administration of TARC–PE38 to HTLV-1-infected humanized mice

Approximately 2 × 10^7^ MT-2 cells were treated with 50 μg/ml MMC (Sigma-Aldrich) at 37 °C for 1 h. Six humanized mice were then inoculated intraperitoneally with ~2.5 × 10^6^ MMC-treated cells per mouse and three control humanized mice were not inoculated. After 2 weeks, either 10 μg of TARC–PE38 per mouse or the same volume of PBS (control) was administered intravenously every day for 4 days into three mice per group inoculated with MT-2 cells, and three control mice were untreated. PBMCs were isolated from all nine mice on days 0 (the day of inoculation with MT-2 cells), 14, 21, 28, and later. The frequency of HTLV-1-infected cells and their PVLs were then tested with flow cytometry and quantitative PCR, respectively.

### Statistical analysis

The data were expressed as means ± standard deviations (SD) for statistical analysis. Differences between groups were determined with Student’s *t* test. The correlation coefficient, R, was calculated with Spearman’s rank correlation test to examine the correlation between two parameters. P < 0.05 was considered significant. These analyses were performed using GraphPad Prism (GraphPad Software, La Jolla, CA, USA).

## Additional files

Additional file 1:
**Figure S1.** Viability of MT-2 cells treated with MMC. After MT-2 cells were treated with 50 μg/ml MMC, they were cultured in vitro and the number of live cells was counted daily with the standard Trypan-blue exclusion method to check the viability of MT-2 cells. At three days post-MMC treatment most of the cells were dead. Additional file 2:
**Figure S2.** Phenotype of MT-2 cells. Expression of the indicated molecules on MT-2 cells was analyzed by flow cytometry. This analysis showed that MT-2 cells are CD45^−^CD3^−^CD4^+^CD8^−^. This phenotype of cells was analyzed similarly in samples from humanized mice inoculated with MT-2 cells and data obtained are shown in Additional file 3: Table S1. Additional file 3:
**Table S1.** Detection of MT-2 cells in humanized mice at 2 weeks post-inoculation with non- or MMC-treated MT-2 cells. Frequencies of MT-2 cells and HTLV-1 PVLs were tested with flow cytometry and quantitative PCR, respectively, in splenocytes and PBMCs obtained from humanized mice at 2 weeks post-inoculation with non- or MMC-treated MT-2 cells. In either mouse group MT-2 cells were not detected by flow cytometry (see Additional file 2: Figure S2 also). While the provirus was detected in splenocytes alone obtained from mice inoculated with non-treated MT-2 cells, it was not at all in splenocytes and PBMCs obtained from mice inoculated with MMC-treated MT-2 cells. Additional file 4:
**Figure S3.** Therapeutic effects of TARC–PE38 on CD4^+^CCR4^+^ cells in HTLV-1-infected humanized mice. To assess the therapeutic effect of TARC–PE38 on HTLV-1-infected cells, peripheral blood was obtained from all the mice. The PBMCs were isolated and the frequency of human CD4^+^CCR4^+^ cells was determined with flow cytometry. The data are representative of each group as follows: none, uninfected untreated mice; MT-2 + PBS, HTLV-1-infected mice treated with PBS; MT-2 + TARC–PE38, HTLV-1-infected mice treated with TARC–PE38. The number indicated in the upper right quadrant is the frequency of human CD4^+^CCR4^+^ cells (%). Additional file 5:
**Table S2.** Characteristics of HTLV-1-infected individuals from which blood samples were collected to test a correlation between PVLs and expression of furin. Additional file 6:
**Figure S4.** Independence of furin expression from induced expression of HTLV-1 Tax. Although expression of Tax was definitely induced by adding Cd^2+^ to JPX-9 cells (lower panel), expression of furin was not increased as compared with that before adding it (upper panel).

## Abbreviations

ADCC: antibody-dependent cellular cytotoxicity
ATL: adult T-cell leukemia
CCR4: CC chemokine receptor 4
FcR: Fc receptor
HAM/TSP: HTLV-1-associated myelopathy/tropical spastic paraparesis
HSCs: hematopoietic stem cells
HTLV-1: human T-cell leukemia virus type 1
IL2: interleukin 2
JSPFAD: Joint Study on Predisposing Factors of ATL Development
mAb: monoclonal antibody
MDC: macrophage-derived chemokine
MFI: mean fluorescence intensity
MMC: mitomycin C
NOG: NOD/SCID IL2-receptor-γ-chain-knockout
NOJ: NOD/SCID Jak3-knockout
PBMCs: peripheral blood mononuclear cells
PBS: phosphate-buffered saline
PE: *Pseudomonas* exotoxin
PMA: phorbol 12-myristate 13-acetate
PVL: proviral load
RT: reverse transcription
SD: standard deviations
TARC: thymus and activation-regulated chemokine
TARC-OFA: TARC-fused oncofetal antigen
TARC-PE38: TARC-fused truncated 38-kDa *Pseudomonas* exotoxin
Tregs: regulatory T cells
