# Supplementary figures and images for: Furin-dependent CCL17-fused recombinant toxin controls HTLV-1 infection by targeting and eliminating infected CCR4-expressing cells in vitro and in vivo
Source: Retrovirology. 2015 Aug 20;12:73. doi: 10.1186/s12977-015-0199-8 (PMC4545545; doi:10.1186/s12977-015-0199-8)

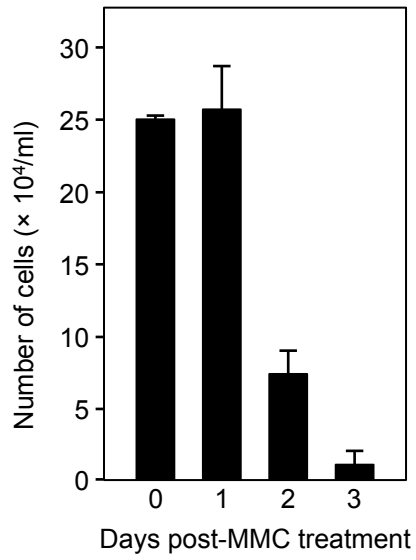

**Figure S1**

Supplement: Additional file 1: — Figure S1. Viability of MT-2 cells treated with MMC. After MT-2 cells were treated with 50 μg/ml MMC, they were cultured in vitro and the number of live cells was counted daily with the standard Trypan-blue exclusion method to check the viability of MT-2 cells. At three days post-MMC treatment most of the cells were dead. [file 12977_2015_199_MOESM1_ESM.pdf]

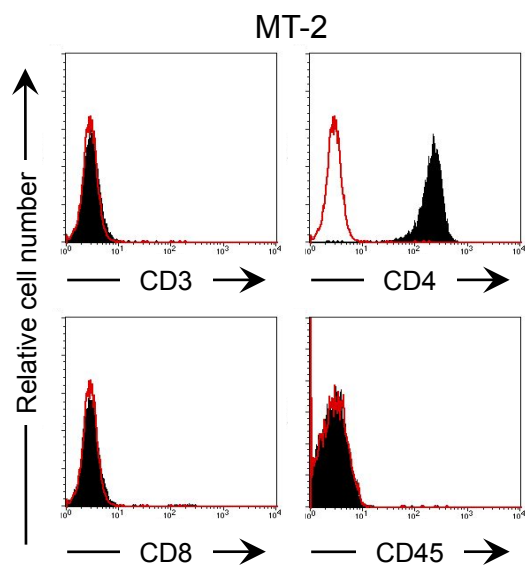

**Figure S2**

Supplement: Additional file 2: — Figure S2. Phenotype of MT-2 cells. Expression of the indicated molecules on MT-2 cells was analyzed by flow cytometry. This analysis showed that MT-2 cells are CD45−CD3−CD4+CD8−. This phenotype of cells was analyzed similarly in samples from humanized mice inoculated with MT-2 cells and data obtained are shown in Additional file 3: Table S1. [file 12977_2015_199_MOESM2_ESM.pdf]

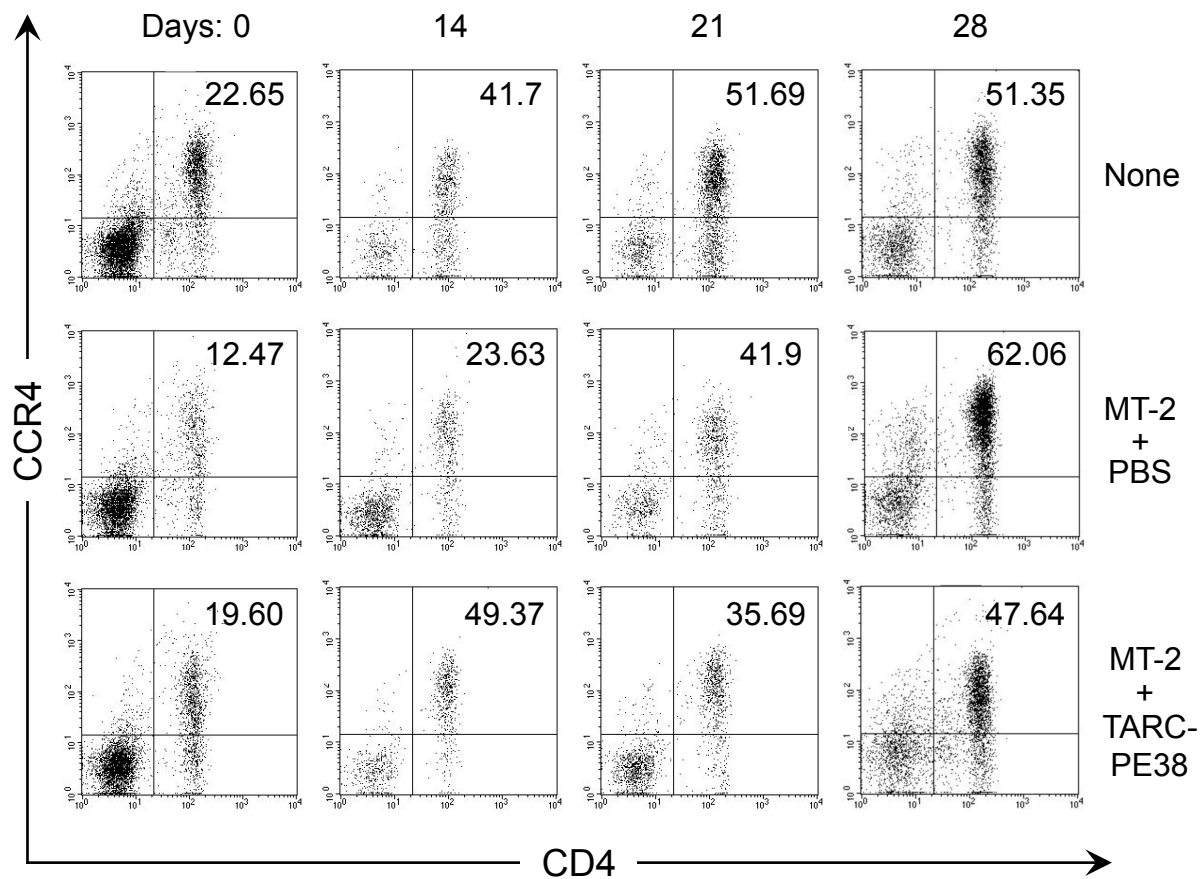

**Figure S3**

Supplement: Additional file 4: — Figure S3. Therapeutic effects of TARC–PE38 on CD4+CCR4+ cells in HTLV-1-infected humanized mice. To assess the therapeutic effect of TARC–PE38 on HTLV-1-infected cells, peripheral blood was obtained from all the mice. The PBMCs were isolated and the frequency of human CD4+CCR4+ cells was determined with flow cytometry. The data are representative of each group as follows: none, uninfected untreated mice; MT-2 + PBS, HTLV-1-infected mice treated with PBS; MT-2 + TARC–PE38, HTLV-1-infected mice treated with TARC–PE38. The number indicated in the upper right quadrant is the frequency of human CD4+CCR4+ cells (%). [file 12977_2015_199_MOESM4_ESM.pdf]

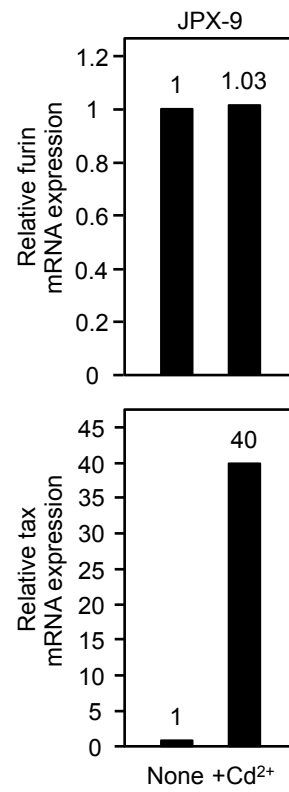

**Figure S4**

Supplement: Additional file 6: — Figure S4. Independence of furin expression from induced expression of HTLV-1 Tax. Although expression of Tax was definitely induced by adding Cd2+ to JPX-9 cells (lower panel), expression of furin was not increased as compared with that before adding it (upper panel). [file 12977_2015_199_MOESM6_ESM.pdf]
