# Supplementary material for: Furin-dependent CCL17-fused recombinant toxin controls HTLV-1 infection by targeting and eliminating infected CCR4-expressing cells in vitro and in vivo
Source: Retrovirology. 2015 Aug 20;12:73. doi: 10.1186/s12977-015-0199-8 (PMC4545545; doi:10.1186/s12977-015-0199-8)
Supplement: Additional file 3: — Table S1. Detection of MT-2 cells in humanized mice at 2 weeks post-inoculation with non- or MMC-treated MT-2 cells. Frequencies of MT-2 cells and HTLV-1 PVLs were tested with flow cytometry and quantitative PCR, respectively, in splenocytes and PBMCs obtained from humanized mice at 2 weeks post-inoculation with non- or MMC-treated MT-2 cells. In either mouse group MT-2 cells were not detected by flow cytometry (see Additional file 2: Figure S2 also). While the provirus was detected in splenocytes alone obtained from mice inoculated with non-treated MT-2 cells, it was not at all in splenocytes and PBMCs obtained from mice inoculated with MMC-treated MT-2 cells. [file 12977_2015_199_MOESM3_ESM.pdf]

Treatment of inoculated MT-2 cells	Mouse No.	MT-2 cells (%)		PVL (copies/100 cells)	
		Splenocytes	PBMCs	Splenocytes	PBMCs
None	1	(-)	(-)	996	(-)
	2	(-)	(-)	1,715	(-)
	3	(-)	(-)	(-)	(-)
	4	(-)	(-)	(-)	(-)
	5	(-)	(-)	885	(-)
MMC	6	(-)	(-)	(-)	(-)
	7	(-)	(-)	(-)	(-)
	8	(-)	(-)	(-)	(-)
	9	(-)	(-)	(-)	(-)
	10	(-)	(-)	(-)	(-)

(-): not detectable

Table S1
