# Supplementary material for: Furin-dependent CCL17-fused recombinant toxin controls HTLV-1 infection by targeting and eliminating infected CCR4-expressing cells in vitro and in vivo
Source: Retrovirology. 2015 Aug 20;12:73. doi: 10.1186/s12977-015-0199-8 (PMC4545545; doi:10.1186/s12977-015-0199-8)
Supplement: Additional file 5: — Table S2. Characteristics of HTLV-1-infected individuals from which blood samples were collected to test a correlation between PVLs and expression of furin. [file 12977_2015_199_MOESM5_ESM.pdf]

Number	Gender	Age	Diagnosis	PVL	Furin
1	Female	65	Carrier	0.31	23.53
2	Female	48	HAM/TSP	34.69	23.05
3	Female	70	Untreated ATL	0.49	17.35
4	Female	62	Carrier	81.54	31.54
5	Male	33	Carrier	2.58	15.21
6	Male	60	Carrier	15.14	24.73
7	Female	55	Carrier	0.23	25.84
8	Male	58	Carrier	21.70	17.88
9	Female	85	Treated smoderling ATL	44.2	30.43
10	Female	55	Carrier	1.51	21.96
11	Male	66	Treated acute ATL	8.92	19.05
12	Female	38	Carrier	0.02	18.89
13	Female	59	Carrier	17.71	27.44
14	Female	30	Carrier	1.04	22.99
15	Female	53	Carrier	0.38	19.18
16	Female	65	Carrier	1.60	17.62
17	Female	44	Carrier	17.21	34.01
18	Female	64	Untreated Chronic ATL	39.82	19.43
19	Female	58	Carrier	123.07	56.09
20	Male	51	Carrier	0.64	13.81
21	Female	51	Carrier	0.38	11.61
22	Female	53	Carrier	0.97	25.83
23	Female	42	Carrier	0.50	19.64
24	Female	60	Untreated Chronic ATL	62.98	23.72
25	Male	38	Carrier	5.98	35.47
26	Female	50	Carrier	2.47	4.80
27	Male	34	Carrier	15.81	21.37
28	Female	37	Carrier	0.67	19.76
29	Female	74	Carrier	0.05	3.10

PVL is presented as copies/100 cells.
Furin is presented as the level relative to that in Jurkat cells.

Table S2
